# Implementing Curcumin in Translational Oncology Research

**DOI:** 10.3390/molecules25225240

**Published:** 2020-11-10

**Authors:** Koraljka Gall Trošelj, Ivana Samaržija, Marko Tomljanović, Renata Novak Kujundžić, Nikola Đaković, Anamarija Mojzeš

**Affiliations:** 1 Laboratory for Epigenomics, Ruđer Bošković Institute, Division of Molecular Medicine, 10000 Zagreb, Croatia; Ivana.Samarzija@irb.hr (I.S.); Marko.Tomljanovic@irb.hr (M.T.); Renata.Novak.Kujundzic@irb.hr (R.N.K.); Anamarija.Mojzes@irb.hr (A.M.); 2 Institute for Clinical Medical Research and Education, University Hospital Centre Sisters of Charity, 10000 Zagreb, Croatia; nikola.dakovic@kbcsm.hr; 3 Department of Clinical Oncology, School of Medicine, University of Zagreb, 10000 Zagreb, Croatia

**Keywords:** cancer therapy, clinical trials, microbiota, IL-17, nicotinamide *N*-methyltransferase, metabolic reprogramming, curcumin formulations

## Abstract

Most data published on curcumin and curcumin-based formulations are very promising. In cancer research, the majority of data has been obtained in vitro. Less frequently, researchers used experimental animals. The results of several clinical studies are conclusive, and these studies have established a good foundation for further research focusing on implementing curcumin in clinical oncology. However, the issues regarding timely data reporting and lack of disclosure of the exact curcumin formulations used in these studies should not be neglected. This article is a snapshot of the current status of publicly available data on curcumin clinical trials and a detailed presentation of results obtained so far with some curcumin formulations. Phenomena related to the observed effects of curcumin shown in clinical trials are presented, and its modifying effect on gut microbiota and metabolic reprogramming is discussed. Based on available data, there is a strong indication that curcumin and its metabolites present molecules that do not necessarily need to be abundant in order to act locally and benefit systemically. Future clinical studies should be designed in a way that will take that fact into consideration.

## 1. Introduction

Therapeutic efforts in the mid 20th century—era preceding smart drugs—were commonly based on very rigid, almost technocratic, attitudes toward many diseases, including cancer. The introduction of highly cytotoxic and non-selectively acting compounds, thought to be potent enough to achieve a complete breakthrough in cancer treatment, did not fulfill early expectations [1]. While the effect of these early synthesized drugs was carefully monitored, they also needed to be continuously modified to improve selectivity and efficacy, while reducing toxic side effects and cross resistance [2]. Even improved versions of originally synthesized and applied compounds have been commonly used with limited benefit for patients and are associated with unavoidable side effects [3].

Numerous observational studies have recorded therapeutic responses varying among patients suffering from the same type of malignant tumor. This reflects heterogeneity that has yet to be fully understood. At minimum, it should be considered on two levels. 

Between-tumor heterogeneity occurs as a consequence of patient-to-patient differences. It can be a major obstacle to predicting a therapeutic response, even in those patients whose tumors express a targetable marker [4]. Well-known examples are breast cancers with amplified receptor tyrosine-protein kinase erbB-2 (HER2/neu) [5]. Within-tumor heterogeneity, resulting from phenotypic and functional cellular diversity within the tumor, directs the outcome of antitumor therapy. This is commonly discussed in the context of therapeutic failure [6]. 

These obstacles in clinical oncology have been a beneficial moving force for encouraging multidisciplinary research efforts to discover compounds which would be not only highly therapeutically effective but also critically selective. New and powerful technologies have been the basis for advances in molecular biology and molecular medicine. In parallel, factual knowledge regarding the precise mechanisms by which certain molecules exert their mode of action became a clinical reality. 

Many processes are very dynamic, including those relevant to tumor development and its response to therapy. They take place during specific time windows during which the host may be affected by varied and numerous external factors.

Currently, we are witnessing therapeutic decisions being guided by an understanding of the molecular features of tumor tissues which develop in the specific genetic background of its host [7]. Even in these situations, in which we appear to be able to precisely determine and characterize the molecular therapeutic target, the side effects of the therapy do not diminish to a satisfactory comfort level.

We are able to study already developed cancer from the point of its mutational status integration into the whole network of aberrant signaling pathways, which are tightly interconnected in the tumor. They are then still highly dependent on the specific parameters of the host itself. Consequently, various cancer features, with their well-known hallmarks, develop as a result of permanent interaction with the host [8]. Diet is of significant influence on these.

Indeed, the influence of diet and nutritional status on cancer occurrence has been investigated and discussed very intensively. While it is well recognized that certain diets definitely are beneficial when facing many diseases, including cancer, some recommendations and observations have yet to be confirmed in properly designed studies [9]. The Mediterranean diet is a good example of a healthy diet. It is based on consuming non-refined cereals, nuts, olive oil, legumes, fish and plenty of fresh fruits and vegetables. With a high content of antioxidative compounds and anti-inflammatory nutrients, the diet may be considered even as chemopreventive with respect to cancer. Still, one cannot mechanistically confirm protective effects of the diet itself just because the dietary pattern produces numerous positive effects during an extended period of time [10]. What all too often remains under the radar is the genetic constitution of the host. 

## 2. Curcumin-Pleiotropic Molecule in a Setting of Personalized Medicine

In this complex setting, which tends to be and should be the basis for a precise and personalized therapeutic approach, it is very difficult to understand the exact molecular mechanism(s) of action of molecules that are pleiotropic. Curcumin, which is used in foods for its typical color, flavor and as a preservative, has been well known for its beneficial effects in traditional medicine for centuries. These days, numerous biological effects were shown to be the bona fide consequence of its three fundamental roles: an antioxidative role when present in low concentration [11], its protein binding properties [12] and its chelating activities [13]. Two of these properties make curcumin yet one more “double-edged sword molecule” because it can also act as a prooxidative molecule when applied in a high concentration [14], and has the potential to decrease systemically available iron [15]. However, the most recent data indicates that curcumin may enhance the effects of low-dose iron supplementation, especially in individuals with iron deficiency, who are otherwise healthy [16].

The natural source of curcumin (diferuloylmethane; IUPAC: (1*E*,6*E*)-1,7-bis(4-hydroxy-3-methoxyphenyl)-1,6-heptadiene-3,5-dione) is the rhizome of the medicinal plant, *Curcuma longa*, a perennial herb in the *Zingiberaceae* family [17]. The yellow color of the rhizome originates from a mixture of three curcuminoides, one of which is curcumin. The content of the commercial turmeric rhizomes can significantly vary as to curcuminoides content, within a range from 0.58% to 6.5% on a dry weight basis [18,19]. It is highly dependent on many factors, including the seed sources, plant age, harvesting and drying processes. The amount and composition of specific curcuminoides in the rhizome also vary. It is commonly reported to be a composition of curcumin (CUR—diferuloylmethane, 77%), demethoxycurcumin (DEM, 17%) and bis-demethoxycurcumin (bis-DEM, 3%) [20]. The stated percentage values are averaged and are considered “standard”.

## 3. Low Bioavailability: How to Understand the Mechanism of Action?

There are numerous studies showing curcumin’s low bioavailability, which is commonly estimated based on its very low level in plasma, when and if detectable. The most important factors, described in a recent review, which explain a low bioavailability, are its poor water solubility, chemical instability, rapid metabolism, short half-life and poor intestinal absorption [21].

Early experiments on the application of curcumin and its bioavailability in rats, showed that the feces contained 75% of the orally applied curcumin [22]. Similarly, oral administration of radiolabeled curcumin led to 89% radioactivity in feces and 6% in urine [23]. The poor bioavailability of curcumin has been one of the major challenges in understanding its mode of action, especially when interpreting the available data on its chemopreventive or beneficial effects in vivo, commonly in animals and rarely in humans. However, the fact that curcuminoides and their metabolites may be present in colon mucosa [24] has opened a quite broad horizon for exploring its effects on gut microbiota, which can manifest as various local and, especially, systemic effects. Its modulatory effect on gut microbiota may, at least in part, explain some interesting phenomena observed and published in the literature. Indeed, as clearly proposed in a recently published review [25], an existing discrepancy between curcumin’s poor bioavailability and chemical instability on the one hand, and convincingly manifested activities recorded in vitro and in vivo on the other hand, deserve to be considered. It is particularly important to understand the relevant aspects of the oral application of curcumin, considering the fact that it affects one complex superorganism, that the human host and its symbiotic microbiome represents [26].

The first record of curcumin application in modern medicine dates back to 1937, when it was used for treating 67 patients suffering from various forms of subacute, recurrent, or chronic cholecystitis [27]. The positive therapeutic response recorded then was the basis for the future interest in curcumin and its healing properties, especially its anti-inflammatory properties. They were among the first studied and, when considered from the perspective of the modern medical era, may be very interesting with respect to several cancer hallmarks, especially for preventing the tumor-promoting inflammation and metabolic reprogramming. Many of its biological roles, including these two, relate to its ability to act as a powerful electron donor and metal ion chelator, as already stated. A strong and well-known activity against reactive oxygen species (ROS) is due to the presence of conjugated double bonds in its chemical structure [28]. Already in 2009, it was shown that its α,β-unsaturated β-diketo moiety makes the chemical basis for metal ions chelation, as shown for iron [29]. 

Although commonly called a “drug” or “therapeutic”, and notwithstanding encouraging data that show its mostly beneficial effects in quite heterogeneous scenarios, as yet, curcumin cannot be and should not be considered any of these. There are many reasons for this, and some of them have been addressed and thoroughly discussed in several recent papers [30,31]. Without wanting to discourage further scientific research and medical therapeutic development involving curcumin, here, we plan to address several levels of inconsistency connected with the clinical trials currently listed, including those relating to the need for timely reporting the data/results [32].

## 4. Current Status of Curcumin-Related Oncological Clinical Studies Listed at ClinicalTrials.Gov

For the purpose of this review, we analyzed the current status of curcumin in cancer translational research during August and September of 2020, at several levels. First, we stratified clinical studies relating to cancer and curcumin currently listed at National Institutes of Health (NIH) clinicaltrails.gov, on the following parameters: the status of the study, the type of cancer and the formulation given. Second, we collected and selected data to be presented for showing the effect of precise curcumin formulations aimed at improving its bioavailability. For selected formulations currently used in clinical trials, the effects obtained in vitro and in animal models are also presented. This set of data is expanded by including previously published studies. A significant portion covers clinical trials and is published in the journals indexed in Web of Science that are also available at PubMed. Third, we discussed the effect of curcumin as presented in new data on the gut microbiota and some exciting aspects of curcumin’s systemic effects. Fourth, we presented curcumin’s possible effect in modifying cancer cell metabolism.

We summarized the status of currently listed clinical trials relating to curcumin in clinical oncology which are available at ClinicalTrials.gov, “a database of privately and publicly funded clinical studies conducted around the world”. The “condition or disease” was defined as “Cancer” and was combined with the word “Curcumin” under “Other Terms”. The search yielded 69 clinical studies. Out of these 69 clinical studies, 10 were not directly related to cancer treatment, but were mainly focused on testing curcumin’s effect in alleviating radiation/chemotherapy side effects. 

Out of 59 studies that were or are, focused on cancer, 11 were excluded from the detailed analyses. Two studies were based on applying turmeric either alone (NCT03061591) or combined with Omega-3+ Vitamin D (NCT03290417). Two studies were based on applying curcumin with various polyphenols (NCT03482401) and anthocyanins (NCT01948661) in breast and colorectal cancer patients, respectively. Three studies were based on applying minimal curcumin and vitamin D in leukemia (NCT02100423), cervical/uterine (NCT03192059) and pancreatic cancer patients (NCT02336087). In the remaining two studies, curcumin was combined with Q10 for myelodysplastic syndrome (NCT00247026) and with fish oils for lung cancer patients (NCT03598309). Two chemopreventive studies (colon) compared the effect of curcumin vs. Sulindac (NCT00176618) and curcumin vs. Sulindac vs. rutin vs. quercetin (NCT00003365).

### The Status of Clinical Studies with Curcumin Only (n = 48)

The remaining 48 studies may be stratified by different criteria, of which the status of the study may be a good indication of the current confusion regarding implementing curcumin into cancer clinical arena. 

So far, 21 studies were indicated as completed (*n* = 21). The status of the remaining 27 studies varies: active, not recruiting (*n* = 5), recruiting (*n* = 4), not yet recruiting (*n* = 3), currently suspended due to COVID-19 (*n* = 1), terminated (*n* = 3), withdrawn (*n* = 4) and unknown status (*n* = 7).

We were particularly interested in the reason for the termination or the withdrawal of studies. For the two studies, the reason for termination was for “futility in view of the results of the interim analysis”, and “for futility in view of the results of the anticipated analysis”. The termination of the third study was attributed to “technology problems and cost constraints”. With respect to withdrawn studies, only one study (NCT00248053) claimed that: “Subsequent data generated by our collaborators have shown efficacy with curcumin and quercetin in 5 patients in a non-placebo-controlled trial”. There is no explanation for withdrawing the remaining three studies. With respect to common claims which tend to present curcumin and its derivatives as potent antitumor agents, this set of data is not optimistic. 

Seven studies are presented with “unknown status”, because the status of the study has not been verified within the past two years. However, after performing an additional search, we were able to find out that one of these studies has published results in a peer-reviewed journal (NCT02138955) [33] and one study (NCT00689195) published results unrelated to curcumin [34]. 

For the rest of the five studies with “unknown status”, we were unable to find published data (NCT00973869, NCT02554344, NCT00295035, NCT00486460, NCT02321293). 

We found that one study (NCT02724618) listed as “active, non-recruiting”, has results published with “nanocurcumin”, which was given to prostate cancer patients undergoing radiotherapy in a randomized, double-blind, placebo-controlled phase II trial. The precise formulation of the compound given: nanocurcumin-SinaCurcumin^®^ used, was not described. No significant difference was shown between the SinaCurcumin^®^ and placebo group with respect to radiation-induced cystitis, hematologic findings and apparent diffusion coefficient (ADC) [35].

Out of 21 studies with the status “completed”, only four have reported results obtained on the ClinicalTrials.gov web page (NCT01740323, NCT00641147, NCT00113841, NCT00365209). Out of these four studies, only one (NCT00641147) reported that results are published in a peer-reviewed journal [36]. The design of that study relied on a pure, 100% curcumin that was taken two times a day for 12 months in a cohort of patients (*n* = 22) who were family members of patients diagnosed with familial adenomatous polyposis (FAP) and having intestinal adenomas. While the treatment itself was well-tolerated, there was no significant difference between the mean number of polyps in the placebo group as compared with the curcumin group. The fact that the intake of 1.5 g curcumin twice daily is well-tolerated does not seem to be a surprise, since the earlier clinical research data showed good tolerance and safety at doses between 4 and 8 g/day, even up to 12 g/day [37]. However, it was very disappointing that there was no effect of curcumin on intestinal adenomas which was the primary target of the trial. Even more so, because previously, Carrol’s study showed a strong effect of curcumin with respect to a decrease in the number of aberrant crypt foci (ACF), which was achieved with oral intake of four grams of curcumin for a month [38]. We were able to find published results from seven other completed studies (NCT01160302, NCT01490996, NCT03211104, NCT03072992, NCT01917890, NCT01712542 and NCT02017353), which, unfortunately, have not reported results on the ClinicalTrials.gov website. The results from the first study (NCT01160302) will be explained in a subsection on microbiota and interleukin 17 (IL-17) [39]. The results from the second study (NCT01490996) were published in three papers, showing the effects of conventional cancer therapy combined with curcumin [40,41,42]. 

The third study, NCT03211104, did not precisely specify the formulation of curcumin (“curcuminoid powder in capsule form”) given to prostate cancer patients with intermittent androgen deprivation in a randomized, double-blind, placebo-controlled trial [43]. Increase in prostate-specific antigen (PSA) during the time of curcumin intake (6 months) was significantly less than in the placebo group (*p* =0.0259). The fourth study (NCT03072992) was based on CUC-1R applied intravenously together with paclitaxel to the patients with advanced, metastatic breast cancer.

The group of patients receiving CUC-1R + paclitaxel had statistically significant improved ORR (overall response rate) when compared with the group receiving paclitaxel + placebo (*p* < 0.01) [44]. 

The results from the fifth study (NCT01917890) were based on applying curcumin formulation BCM-95^®^ to prostate cancer patients being treated with radiotherapy [45]. The sixth study (NCT01712542) was based on giving micellar NovaSol formulation to glioblastoma patients (57.4 mg curcumin, 11.2 mg demethoxycurcumin, 1.4 mg bisdemethoxycurcumin). Curcumin was present in tumorous tissue (average, 56 pg/mg) but its amount significantly varied among tumors (range 9–151 pg/mg) [46].

The seventh, open-label, non-randomized phase 2 study (NCT02017353), was based on the hypothesis that curcumin supplementation might influence inflammatory biomarker levels in patients (*n* = 7) suffering from endometrial carcinoma (EC). The patients consumed Curcumin Phytosome-Meriva® orally, 2 g/day, for 2 weeks. The results of the study were not definitely conclusive [47].

All together, among 48 analyzed studies based on application of curcumin, four studies reported results at ClinicalTrials.Gov., of which one published the data. Among 21 studies with the “completed” status, seven studies, resulting in nine papers, did not report the data at ClinicalTrials.Gov. 

Based on these data, the problem becomes clear: the studies have been designed to explore the efficacy of curcumin being given to patients with various types of premalignant lesions/cancer, using heterogeneous curcumin formulations that are to be given (or were given) in daily doses which significantly differ. There are various application methods utilized during significantly different time periods. As well, the hosts (patients) differ in many respects, especially genetically (between-tumor heterogeneity). A host’s genetics is the only parameter that cannot be changed for improving curcumin clinical trials. Everything else, including the design of the studies and disclosing details on curcumin formulations given, can be and should be improved. 

**Figure 1:**
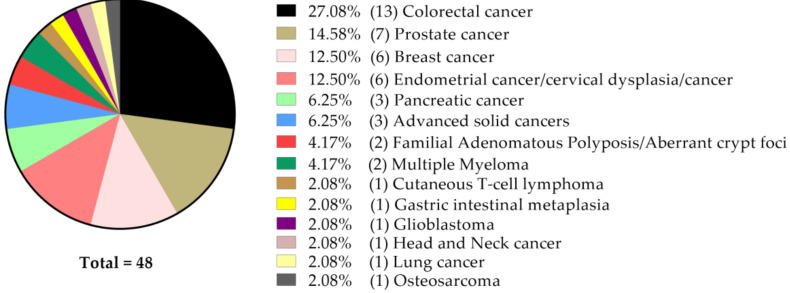
Number and percentage of studies focused on a particular type of cancer, according to its anatomical origin.

With respect to the type of cancer, a majority of the 48 studies focused on colorectal (*n* = 13) and prostate (*n* = 7) cancer, followed by breast (*n* = 6), uterine (endometrium and cervix, *n* = 6) and pancreatic cancer (*n* = 3) models (Figure 1).

**Figure 2:**
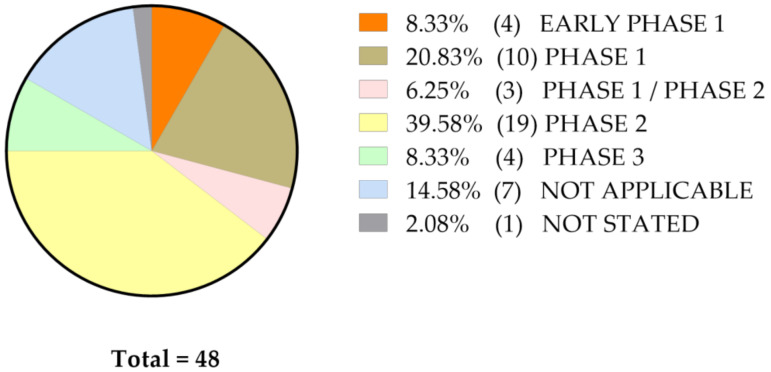
Reported phases of clinical trials involving curcumin, regardless of its formulation and type of malignant disease.

The inclusive criteria and final goals are highly variable, which leads to stratifying these studies in different stages of clinical trials, as shown on Figure 2. 

**Figure 3:**
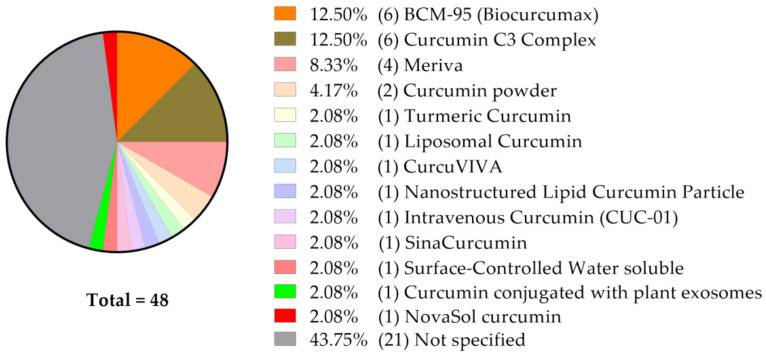
Among 48 clinical studies currently listed at ClinicalTrials.gov and analyzed as described, 43.75% (*n* = 21) do not specify the curcumin formulation used/to be used.

A very high portion of studies do not disclose the precise curcumin formulation given (Figure 3).

## 5. The Effect of Curcumin Formulations Targeted at Improving its Bioavailability

Curcumin Phytosome-Meriva®, the formulation given in the last study described in the previous section, is only one of many curcumin-based formulations targeted at improving its bioavailability. Already in 1998, Shoba et al. described how piperine, the constituent of black pepper, then already known to be an inhibitor of hepatic and intestinal glucuronidation, increased curcumin’s bioavailability in humans by 2000%. This conclusion was based on curcumin’s value in serum [48]. Since then, many formulations were synthesized and tested [21].

As presented on Figure 3, BCM-95^®^ (95% curcuminoid complex with preserved natural ratio of curcumin, demethoxycurcumin, and bisdemethoxycurcumin and essential oil of turmeric [49], Table 1), Curcumin C3 Complex^®^ (reported formulations vary: curcumin: 77–90%, demethoxycurcumin: 6–17%, bis-demethoxycurcumin: 3–4% [50,51], Table 2) and Meriva^®^ (curcumin, soy lecithin, microcrystalline cellulose, 18–20% curcuminoids [52], Table 3) appear to be the most commonly explored in the clinical trials we followed. For that reason, we have decided to present as much information as possible on these formulations when applied in vitro, in animal models and when tested in clinical trials.

We extended our search to three currently promising formulations: liposomal curcumin (Lipocurc™, Table 4), the blood-brain barrier crossing Longvida^®^ (solid lipid curcumin particle, phosphatidylcholine and 20% curcumin [53], Table 5) and a colloidal nanoparticle-based formulation of curcumin, Theracurmin™ (THC; 10% *w*/*w* of curcumin, 2% of other curcuminoids such as demethoxycurcumin and bisdemethoxycurcumin, 46% of glycerin, 4% of gum ghatti and 38% of water [54], Table 6), which was shown to exert a very strong antiproliferative effect, especially in combination with NAD(P)H quinone dehydrogenase 1 (NQO1) inhibitor [55]. For all formulations but Lipocurc™, the pharmacokinetics data obtained in humans were recently reviewed [56,57]. For Lipocurc™, the available data on humans indicates stable curcumin plasma concentrations during infusion, and soon thereafter, a rapid decline to an undetectable level [33]. 

It is well-known that curcumin may improve the therapeutic effect of many tested drugs. We have written about that earlier in the context of its inhibitory effect on nuclear factor kappa-light-chain enhancer of activated B cells (NF-κB) [58], in combined cancer therapy. Here, we present combined treatments for cancer with the earlier listed formulations in vitro, in animal models and in clinical studies. Case reports are not presented. Papers in which formulations were not specifically disclosed are not included in the tables presented here. Although the U.S. Food and Drug Administration (FDA) considers curcumin as a GRAS (Generally Recognized as Safe) molecule, some side effects were observed in clinical studies when higher doses were taken. These adverse effects are also shown in the tables.

## 6. Microbiota and Curcumin: Acting Locally Benefiting Systemically?

It is now well-established that curcumin can influence intestinal microflora directly [118] and indirectly through the effect of active curcumin’s metabolites on metabolic activity of gut microbiota. The major processes included are demethoxylation, reduction, hydroxylation, methylation and acetylation of the parent compound. This was shown for the first time in 2015, when 23 metabolites were characterized after incubating human stool with 100 μM curcumin [119]. There are many research results showing curcumin’s effect on gut microbiota in rats, mice and humans. However, it has to be clearly stated that one must avoid making any kind of parallel between curcumin’s effect on gut microbiota based on results obtained in different species, because there is a significant difference in the relative abundance of major gut bacterial families and genera in mice, rats, non-human primates and in humans [120]. 

A prospective, single-center, evaluator-blinded randomized pilot study including a total of 32 adults, showed that turmeric/curcumin influences the content of gut microbiota in healthy humans in a highly personalized way. However, changes in the pattern of microbial species abundance were highly similar in a turmeric group (1 g turmeric root—6 g daily (*Curcuma longa*) plus 1.25 mg black pepper-derived extract of piperine alkaloid (BioPerine)) and a curcumin group (combination of C3—6 g daily, and BioPerine). This suggests that curcumin indeed has a major influence on microbiota composition alterations. An increase of *Clostridium* spp. and *Bacteroides* spp. was particularly strong [121]. An increase of *Bacteroides* spp. was also shown in mice fed a curcumin diet [122]. 

In another mouse model, in which animals were fed a diet containing 0.2% (*w*/*w*) nanoparticle curcumin—Meriva®, the clinical presentation of experimentally induced inflammatory bowel disease (IBD) was significantly improved and was associated with an increase of *Clostridium* cluster IV and *Clostridium* subcluster XIVa. The stool contained a high level of one anti-inflammatory short-chain fatty acid (SCFA)—butyrate. The content level of CD4+ Foxp3+ regulatory T cells and CD103+ CD8α− CD11c+ dendritic cells in intestinal mucosa was high [123]. These findings agree with previously published research papers, unrelated to curcumin, showing butyrate (a) as an inducer of Treg cell differentiation in colon, which is crucial for suppressing inflammatory and allergic responses [124], and (b) as a suppressor of colonic epithelial stem/progenitor cell proliferation [125]. Thus, it seems that some beneficial effects of curcumin obtained locally and systemically, with respect to various pathological conditions, may indeed be related to its effect on butyrate-producing gut bacteria. 

This may be of a particular relevance for appreciating and understanding the beneficial effects of curcumin observed in colon cancer patients, known since 2005 [91]. We can now consider those data from a very interesting perspective, relating to curcumin’s effect on microbiota. Earlier this year, Sánchez-Alcoholado et al. have shown that colon cancer patients have significantly decreased abundance of *Bacteroidetes* [126]. If curcumin can induce increased abundance of *Bacteroides* spp. (which should be in accord with Reference [121]), then maybe the butyrate potentially benefited the participants in the study published back in 2005 [91]. 

In our recent paper, we considered the importance of curcumin with respect to interleukin-17 (IL-17)-related effects in humans, especially in the field of oncology [127]. We discussed the fact that curcumin strongly decreases the serum level of IL-17 at one hour post-ingestion of C3 Complex^®^ (*p* = 0.0342), as shown in 15 head and neck squamous cell carcinoma patients and eight healthy volunteers [39]. The possible reason for IL-17 decrease may well be the curcumin’s effect on gut microbiota. 

A mouse model of experimental colitis was explored by Zhao and collaborators, showing that activation of intestinal dendritic cells by curcumin leads to enhanced suppressive functions of Treg cells joined with a decrease in several mediators of inflammation in colonic mucosa, including IL-17. Of note, the decrease was measurable, but did not reach statistical significance [128]. In humans, using an organ culture chamber, Larussa et al. have shown, based on 35 human gastric biopsies, that 200 μM curcumin significantly decreases IL-17 in both gastric biopsies (*p* = 0.0003) and culture supernatants (*p* = 0.0001) [129]. The mechanism described may facilitate the persistence of the bacterium locally due to a decreased inflammatory reaction [129]. However, one may ask whether decreased IL-17 production in the colon, as a consequence of the presence of curcumin/curcumin’s metabolites, may also be beneficial for the host, due to the possible influence even on such harmful systemic effects as hypertension [130], mediated by IL-17. 

It is well-known and was recently reviewed in depth that the role of IL-17 in cancer may be suppressive or promotive [131]. For that reason, the precise role of this cytokine, in a well-defined cellular setting, should be entirely ascertained before coming to a firm conclusion on curcumin’s role through modulation of IL-17 secretion. 

There are data showing that gut microbiota depletion by antibiotics leads to a significant decrease in subcutaneous tumor burden in pancreatic cancer and melanoma models in mice. This is only in animals having mature T and B lymphocytes [132]. In those animals, there was a significant increase in anti-tumor interferon gamma(IFNγ)-secreting T cells (IFNγ + CD3+), associated with a decrease in IL17a (IL17a + CD3+) and IL10 (IL10 + CD4 + CD3+) secreting immune populations. If the animals were treated with IL-17a neutralizing monoclonal antibody, the tumor-attenuating effect of antibiotics was abrogated. Thus, at least in this mouse model, the essential role of IL-17a in tumorigenesis seems to be clearly confirmed. 

**Figure 4:**
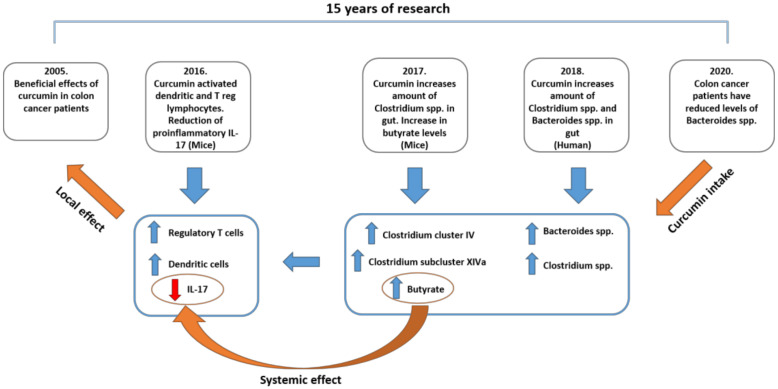
A proposed model for explaining systemic effects consequential to curcumin’s local activity. The local effect obtained in the clinic back in 2005 can be explained through recent findings on gut microbiota.

Based on all these data, especially on the very recent data, it appears that curcumin does not need to be measurable in plasma in order to exert an effect. It has the ability to modulate the gut microbiota and reprogram differentiation of crucial T-cell population(s) in the gut. Thus, indeed, it may have the potential to act locally while also reaching numerous targets systemically (Figure 4).

## 7. Curcumin in Targeting Metabolic Liabilities in Cancer

Metabolic heterogeneity needs to be considered in the context of the developmental program of epithelial-to-mesenchymal transition (EMT), which allows cancer cells to acquire features needed for their survival. These include increased migratory potential, an ability to invade tissues around the primary tumor, extravasation into lymphatics or blood vessels and migration to distant sites in the body [133]. This factor significantly contributes to microenvironment-mediated metabolic heterogeneity of cancer tissue. Proliferation of disseminated cancer cells at distant sites is possible because the migrated cells pass through a mesenchymal-to-epithelial transition (MET) once they reach their destination [134]. Metabolic reprogramming in cancer, involving an increase in glucose uptake, glycolytic flux and lactate production, contributes to the acquisition of cancer stem cell properties and tumor aggressiveness [135]. This takes place by an orchestrated activity of expressing EMT-inducing transcription factors (TF) and their transcriptional targets, some of which are metabolic enzymes.

In 2006, the suppressive effect of curcumin on transcriptional activity of HIF1A gene under hypoxia was shown [136]. The protein product coded by this gene, Hypoxia-Inducible Factor 1-Alpha-HIF1α, is a transcription factor, central to metabolic reprogramming in cancer and EMT-induction [137]. 

HIF1α contributes to EMT at many levels, which can be significantly affected with pleiotropic curcumin’s action. For example, through silencing of HIF1A, curcumin can silence expression of its transcriptional target hexokinase 2 (HK2), the enzyme that catalyzes the first step of glycolysis (phosphorylation of glucose to glucose-6-phosphate). Increased expression of HK2 under hypoxia directed by HIF1α has been reported to promote EMT in tongue squamous carcinoma [138]. In addition to through HIF1α, curcumin can influence the multiple, cell-type-specific transcriptional control of HK2 by decreasing the activity of STAT3 [139] and snail family transcriptional repressor 2-SLUG. In 2016, the existence of this second scenario was shown in breast cancer cell lines [140]. Dissociation of HK2 from the colon cancer cells mitochondria through an unknown mechanism was also shown [141].

Pyruvate kinase isoform PKM2, which catalyzes the final step of glycolysis and promotes the Warburg effect, and is a positive regulator of STAT3 activity [142], has been shown to promote EMT. Nuclear translocation of PKM2 and its direct interaction with a transcriptional cofactor repressor TGF-β-induced factor homeobox 2 (TGIF2), causes recruitment of histone deacetylase 3 to E-cadherin promoter, leading to EMT through suppression of E-cadherin transcription [143]. Curcumin has been shown to downregulate the expression of PKM2 [144,145] and to influence its nuclear localization, albeit in a cell-type-specific manner [145]. In line with curcumin’s negative effect on PKM2 expression and nuclear localization, upregulation of E-cadherin upon curcumin treatment has been reported in various types of cancer cells [146,147,148,149]. Concomitant with reducing HIF1α expression, curcumin reduces the expression of EMT-inducing factors Twist Family bHLH Transcription Factor (TWIST) and Zinc Finger E-Box Binding Homeobox 1 (ZEB1) [150], since HIF1α-binds to hypoxia-response elements (HRE) in their promoters [151,152].

In addition to hypoxia, nutrients availability has a profound influence on the interdependent processes of metabolic and epigenetic reprogramming in cancer [153]. Prolonged glucose deprivation has been shown to contribute to the phenotypic plasticity of ovarian serous carcinoma cells (OSCS), characterized by acquiring stem cell properties and resistance to glucose restriction, dependent on the expression of nicotinamide *N*-methyltransferase (NNMT) [154]. This enzyme methylates nicotinamide (NAM) and impairs methylation of histones and other proteins in cancer cells [155]. The increased expression of NNMT was shown in numerous, various malignant tumors [156]. Its active presence induces epigenetic and metabolic reprogramming for promoting the mesenchymal phenotype of cancer cells through increasing migration, invasion, proliferation and survival. On the other hand, knockdown of NNMT suppresses cancer cell migration and EMT. NNMT was shown to be a critical regulator of EMT in esophageal cancer cell lines and was suggested to be a potential therapeutic target for metastatic squamous cell carcinomas [157].

NNMT is under the transcriptional control of STAT3, which is often overexpressed in various types of cancer. Curcumin, by direct binding to cysteine residue 259, prevents STAT3 phosphorylation, dimerization and translocation into the nucleus, as shown in H-Ras-transformed breast epithelial cells, H-Ras MCF10A [158].

In the cancer cells which rely on STAT3-mediated increase of NNMT expression, this can be of the utmost importance. NNMT is thought to be “a poor prognostic biomarker for patients with solid tumors”, based on a meta-analysis performed on 3340 patients [159]. If the cells lose NNMT, as a result of curcumin’s action, then they lose a powerful oncogenic player—a master metabolic regulator of cancer-associated fibroblasts (CAF)—as shown in high-grade OSCS [160]. In line with the negative effect of curcumin on STAT3 activity, downregulated expression of NNMT was shown in vitro, in MDA-MB-468 breast cancer cells and HT29 colon cancer cells [161].

The activity of NNMT promoter is regulated in a complex, tissue-specific fashion [162]. Keeping in mind the importance of the cell-type-specific modes of transcriptional control, it deserves to be explored in depth in which types of tumors curcumin exerts its negative effect on EMT through STAT3-mediated silencing of NNMT, as well as HK2. 

**Figure 5:**
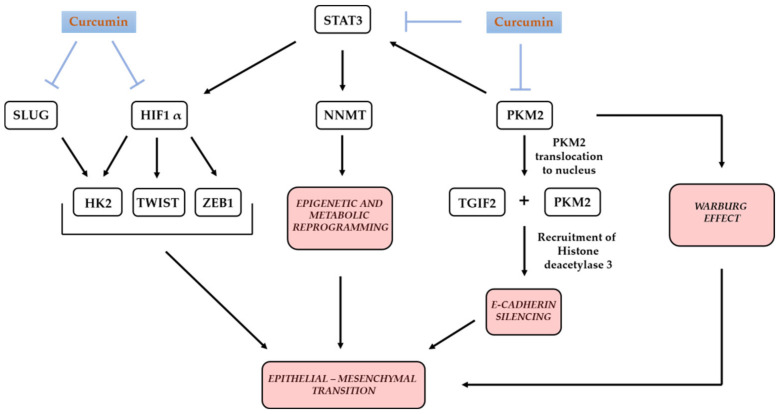
Simplified presentation of an inhibitory effect of curcumin on key molecular players at the cross-roads of epithelial-to-mesenchymal transition and metabolic reprogramming in cancer cell.

A simplified presentation of some aspects of curcumin’s modifying effects on metabolic reprogramming and EMT is shown in Figure 5.

### Curcumin-Mediated Suppression of NNMT and Other Metabolic Enzymes in Circumventing Therapy Resistance

The connection between nutrient status, NNMT expression and its role in EMT should be considered with respect to cancer therapy. It has been recently reported that the expression of NNMT in human esophageal squamous cell carcinoma cell lines decreases sensitivity to 5-Fluorouracil (5-FU) by promoting the Warburg effect. In a cell line with high NNMT expression (TE1 cells), the sensitivity was low, while the highly 5-FU-sensitive EC1 and Eca109 cells had a low NNMT expression. Downregulation of NNMT in TE1 cells increased their sensitivity to 5-FU, accompanied by lower glucose consumption, lactate production, and the expression of hexokinase 2, lactate dehydrogenase A and phosphoglycerate mutase 1 [163]. Curcumin was shown to increase the sensitivity of cancer cells to 5-FU through the silencing of NF-κB [58]. Considering its pleiotropism and ability to modify activity of numerous cellular molecules, it is conceivable that enhanced sensitivity to 5-FU by curcumin (Table 1 and Table 2) may be a direct consequence of its silencing effect on the axis STAT3/NNMT, and not only NF-κB and its targets. Decrease in the strength of the axis STAT3/HK2, which was recently unquestionably shown as a mechanism responsible for 5-FU resistance in HNSCC [164], may also be the mechanism with which curcumin increases sensitivity to conventional chemotherapy in vitro [61,66,67,68,72,84,96].

Finally, some metabolic enzymes were shown to be not only crucial for sustaining cancer cells’ metabolic needs, but also for development of therapy resistance. For example, phosphoglycerate dehydrogenase (PHGDH), the rate-limiting enzyme of de novo serine biosynthesis, is commonly overexpressed in malignant tumors and was recently shown to promote bortezomib resistance of multiple myeloma cells [165]. Serine hydroxymethyltransferase (SHMT2) catalyzes the conversion of serine to glycine, and is essential to cell survival under hypoxic condition, when it is under transcriptional control of HIF-1α [166]. Silencing of SHMT2 increases sensitivity of hepatocellular cancer cells to doxorubicin [167]. To which extent curcumin’s direct binding to these two enzymes [12,168] contributes to previously shown reversion of the chemoresistant phenotypes related to these two drugs [169] remains to be explored.

## 8. Conclusions

Curcumin’s influence on energy metabolism and stress-response-relevant proteins is tightly linked to redox status in cancer [170]. Curcumin’s activity is mediated by its oxidative metabolites. The varying extent of its redox-dependent bioactivation in diverse in vitro and in vivo models contributes to the current inconclusive results regarding its influence on a particular target [171]. Still, its beneficial effects shown in studies performed in vitro, in vivo and in clinical trials should be considered from as many perspectives as possible. The varying effects of curcumin, which could be due to its metabolites, are a potential advantage, applied in the proper context [172]. The major obstacles to obtaining consistent and measurable results when studying curcumin’s effect in the context of a specific cancer cell line in vitro or cancer in vivo are accentuated by the numerous variables in a vivid biological system. They are inherent in the tumor cell tissue of origin, are driven by oncogenotype (mutations in different cancer-relevant genes result in different phenotypes of tumors arising in the same tissue) and arise by the interaction with tumor microenvironment (vascularization-dependent nutrient and oxygen availability, local nutrient milieu, dependent on non-malignant cells and host microbiome) [173]. It is conceivable that this metabolic heterogeneity and flexibility makes cancer treatment, including targeting its metabolic liabilities, difficult to treat [174]. Still, there are numerous excellent research data which should accompany double-blinded, placebo-controlled clinical trials, and become a moving force for ever more fruitful research and eventual therapy. For meaningful basic medical science, curcumin’s effects need to be critically measured, presented and put in the context of findings that do not necessarily relate to curcumin research. The scope for understanding the way of acting of a pleiotropic molecule must be very broad. At the same time, several aspects of clinical trials nesed to be improved so that these efforts can be applied toward the particular cancer and other targets needing reliable attention.

## Figures and Tables

**Table 1:** Effects of BCM-95^®^ (Biocurcumax™) obtained in vitro, in vivo and in clinical trials.

BCM-95^®^ (Biocurcumax™)		
**MONO**		
**Cell Culture Models**	**Animal Models**	**Clinical Studies**
Suppression of cross-talk between HCT-116 colon cancer stem cells and MRC-5 fibroblasts in the 3D co-culture model [59]. Cytotoxic effect on HCT116 and SW480 colon cancer cell lines; promotion of cell-cycle arrest and apoptosis, strong upregulation of hsa-miR 34a and enhanced expression of F-Box and WD Repeat Containing 7 (FBXW7) [60]. A strong effect on cellular viability and colony formation ability of pancreatic cell lines (BxPC3-G and Panc1), already as 5 μM [61].	HCT116-5-FUR (5-fluorouracil resistant) xenografts in nude mice (50 mg BCM-95^®^/kg daily i.p. for 40 days): attenuation of tumor growth [62]. HCT116 xenografts in mice (25 mg BCM-95^®^/kg daily i.p.; 20 days): enhanced suppression of tumor growth; decrease of miR-27a; increase of miR-34a [60]. The BxPC3-G xenografts in athymic mice (100 mg BCM-95^®^/kg daily i.p.; 28 days): suppression of tumor growth (*p* < 0.05) downregulation of plasmocytoma variant translocation 1 (PVT1), Myc and multidrug resistance-1 (MDR1) [61].	A double-blinded, randomized, placebo-controlled study (*n* = 40) involving prostate cancer patients being treated with radiotherapy. Giving 3 g of BCM-95^®^ starting 1 week before the initiation of radiotherapy and continuing until its completion: no significant effect on treatment outcome, but recordable effect on oxidative status during and after radiotherapy, presented as increased total plasma antioxidative activity and decreased activity of plasma superoxide dismutase (SOD) [45]. A randomized double-blind placebo-controlled phase II B trial, (*n* = 223) involving 111 patients with oral leukoplakia + 121 patients in the placebo group. Giving BCM-95^®^ (3.6 g/day) for six months was well-tolerated and demonstrated significant (*p* = 0.02) and durable clinical response for 6 months [63]. Double-blind placebo randomized-controlled trial (*n* = 121) involving patients with cervical cancer being treated with radiotherapy. Giving 1 g of BCM-95^®^ orally, three times daily for 9 weeks: there was no evidence of improved clinical response to radiation treatment [64]. A randomized, blind placebo-controlled trial (*n* = 61); patients receiving radiotherapy for head and neck squamous cell cancer and receiving three daily doses of 500 mg BCM-95^®^ CG capsules; total daily dose 1.5 g until radiotherapy completion: decreased incidence and severity of radiation-induced mucositis, *p* < 0.001 [65]. **Adverse Events (AE):** A total of 61 (27.4%) subjects experienced at least one AE during the study period, 26 (23.4%) in curcumin arm, and 35 (31.3%) in placebo arm. Four subjects from curcumin arm and 1 from placebo arm withdrew from the study due to Adverse Events (AEs)/Serious Adverse Events (SAEs). Moderate/severe AEs were recorded in four patients in the curcumin group: anemia, skin/subcutaneous tissue disorders and hypertension. The Independent Data and Safety Monitoring Board did not identify any safety concerns, as there was no statistically significant difference in major AEs between the curcumin and placebo arms [63]. The most common adverse events were gastrointestinal nausea/vomiting (30% curcumin vs. 24.5% placebo), stomatitis (20% curcumin vs. 25% placebo), diarrhea (3.33% curcumin vs. 0% placebo), increased liver enzymes: aspartate aminotransferase, AST (5% curcumin vs. 3.3% placebo) and alanine aminotransferase, ALT (5% curcumin vs. 0% placebo) and increased creatinine (5% curcumin vs. 1.6% placebo). However, no statistically significant difference between treated and placebo group was found [64]
**COMBINED**		
**Cell Culture Models**		**Animal Models**
Chemosensitization to 5-FU (5-fluorouracil) of DNA mismatch repair (MMR)-deficient and MMR-proficient HCT116 colon cancer cell line through targeting the cancer stem cells subpopulation and the inhibition of β-catenin and NF-κB signaling pathways [66]. Moderate chemosensitization of 5-FU resistant colon cancer cell lines, HCT116R and SW480R, through suppressive micro-RNA (miR-34a, -200c, -141, -429) mediated inhibition of epithelial-to-mesenchymal transition (EMT). In 5-FU resistant cell lines, the combined treatment with BCM-95^®^ increased the rate of apoptosis (10 µM 5-FU, 10 µM curcumin) with respect to 5-FU alone, through upregulated p21, and altered cell cycle through downregulated cyclin D1 (CycD1) and c-MYC [62,67]. Chemosensitization of MMR-deficient and MMR-proficient HCT116 colon cancer cell line to 5-FU in a 3D alginate mimicking tumor microenvironment [68]. Chemosensitization of pancreatic cancer cells (BxPC3-G and Panc1) to gemcitabine by attenuating Polycomb Repressive Complex 2 (PRC2) subunit Enhancer of Zeste 2 (EZH2), and the long non-coding RNA Plasmacytoma Variant Translocation 1 (PVT1) expression [61]. Primary culture of oral cavity cancer cells: decrease of CD44+ population treated with BCM-95^®^ (*p* = 0.0001), and combination of curcumin and metformin (45.7%, *p* = 0.0001). The treatment also inhibited the migratory and self-renewal properties of cancer stem cells (CSC) [69]. Combination of oligomeric proanthocyanidins (OPC) and BCM-95^®^: strong anti-tumorigenic effect shown in six colorectal cancer cell lines. Decrease of tumor organoid formation and growth of patient-derived colorectal cancer cell. Combined treatment, as contrasted with individual treatments, strongly influenced Hedgehog signaling pathway and pathways related to insulin and peroxisome proliferator-activated receptors PPAR [70].		HCT116-5-FUR xenografts in nude mice: 20 mg/kg every 2 days of 5-FU; 50 mg/kg daily BCM-95^®^ i.p. for 40 days; attenuation of tumor growth, indicating significance for re-sensitization against 5-FU [62]. The BxPC3-G xenografts in athymic mice (100 mg BCM-95^®^/kg daily i.p.; 25 mg gemcitabine/kg body weight once every 4 days for 28 days): significant decrease of tumor weight (*p* < 0.01) [61]. 4-nitro quinoline-1-oxide (4NQO) induced murine oral carcinogenesis model (drugs in drinking water: BCM-95^®^ 14.3 μg/mL; metformin: 5 mg/mL for 8 weeks): the combination regimen decreased tumor volume (*p* = 0.0431) and expression of NF-κB and pS6. Improved overall survival with downregulation of the number of cancer stem cells [69]. Athymic mice with subcutaneous xenografs of HCT116 cells: the combination of OPCs and curcumin (both agents at 100 mg/kg) was more potent in decreasing tumor growth than the individual agents (*p* = 0.0006 for tumor volume, *p* =0.000214 for tumor weight) [70].

**Table 2:** Effects of C3 Complex^®^ obtained in vitro, in vivo and in clinical trials.

Curcumin C3 Complex^®^		
**MONO**		
**Cell Culture Models**	**Animal Models**	**Clinical Studies**
The treatment with 40 μM C3 Complex^®^ for 72 h induced autophagy, but not apoptosis in glioma U87-MG and U373-MG cell lines. The mode of action was highly dependent on the status of AKT [71]. Decreased viability of several human multiple myeloma cell lines [72]. Activation of 5-AMP (Adenosine 5′-Monophosphate)-Activated Protein Kinase (AMPK) and suppression of gluconeogenic gene expression in hepatoma cell lines of rat (H4IIE) and human origin Hep3B [73]. Inhibition of carcinogen and nicotine-induced Mammalian Target of Rapamycin (mTOR) pathway activation, cell proliferation, migration and invasion in several head and neck squamous cell carcinoma (HNSCC) cell lines [74]. Inhibition of breast stem cells self-renewal and Wnt signaling. Significant reduction of Aldehyde Dehydrogenase 1 Family Member A1 (ALDH-1A1) expressing cells (from 7.3% to 1.5%) achieved during 72 h with 10 μM C3 Complex^®^ [75]. Induction of differentiation of myeloid-derived suppressor cells (MDSCs) and inhibition of their interaction with MKN-45 gastric cancer cells with consequential decrease of the growth advantage of cancer cells acquired from the interaction with MDSCs [76]. Antiproliferative effect through suppression of Signal Transducer and Activator of Transcription 3 (STAT3) activation in non-tumor-derived, immortalized human bronchial epithelial cells (AALE) and human lung adenocarcinoma cell line H441 [77]. A strong inhibitory effect on the aggressive skin cancer cell line SRB12-p9 presented on the second day of treatment at 20 μM C3 Complex^®^ through an inhibitory effect on pAKT, pS6, phosphorylated Eukaryotic Translation Initiation Factor 4E-Binding Protein 1 (*p*-4EBP1), pSTAT3 and phosphorylated Extracellular Signal-Regulated Kinases 1 and 2 (pERK1/2) [78]. The treatment (20 µM C3 Complex^®^ or vehicle −0.1% dimethyl sulfoxide (DMSO); control) for 48 h induced change of expression of several miRNAs tested. A strong increase of miR-205-5p was recorded in murine melanoma B78H1 cell line and human melanoma SK-MEL-28 cell line, but not in lymph node metastasis human MeWo cell line [51]. Inhibition of UV-B/FGF-2/mTOR–induced proliferation, progression and colony formation of murine epithelial JB6 cells [79]. Already 5 μM C3 Complex^®^ inhibits formation of primary mammosphere (breast cancer cell lines and breast cells isolated from voluntary mammoplasty patients). A strong decrease of transcriptional activity of genes associated with breast stemness was observed [80]. Protection of rat liver epithelial cell line T51B against iron-related neoplastic cell transformation but only when applied in high concentration [81]. Reduction of tumorigenic properties of mesothelioma cell lines (MSTO-221H, NCI-H2452, Ist-Mes-2) by impairing cellular self-renewal ability and decrease of proliferation/migration but only when combined with Bioperine* [83].	Several ovarian carcinoma cell lines xenografts in nude mice; C3 Complex^®^: oral gavage of 500 mg/kg for maximally 6 days, starting one week after inoculation. C3 Complex^®^ monotherapy: significant decrease of tumor weight (55%; *p* = 0.01) in the HeyA8 docetaxel-sensitive model and reduction in tumor burden in the HeyA8 docetaxel-resistant model, when compared with controls (47%; *p* = 0.05) [84]. Xenografts of U87-MG glioma cell line in nude mice: C3 Complex^®^ (100 mg/kg in DMSO in PBS) was given intratumorally every 24 h for 7 days. After 16 days, the size of the tumors in curcumin-treated animals were significantly smaller than in control animals (3.5 ± 2.8-fold versus 12.5 ± 5.9-fold; *p <* 0.05) and was associated with strong induction of autophagy [71]. Sensitization of human colorectal HCT116 cancer xenografts in mice (C3 Complex^®^ 1 g/kg, once daily orally, one week after implantation of cancer cells) to γ-radiation by targeting NF-κB and NF-κB-regulated genes [85]. MAC16 colon tumor-bearing mice (C3 Complex^®^ 250 mg/kg, daily, 21 days): prevention and/or reversion of cachexia through the inhibition of chymotrypsin-like proteasome 20S activity [86]. SCC40 xenografts in Balb/c nu/nu mice: a) treatment (daily oral gavage of 5, 10, or 15 mg in corn oil for 24 days: day 0: the day when tumors reached 40 mm^3^); b) chemoprevention: C3 Complex^®^ application for 4 days (same dosage and way of application as under “a“), prior to grafting. Highly effective in both suppressing tumor growth and initiation, the activity is associated with modulation of various signaling pathways, including mTOR’s downstream target, ribosomal protein pS6 [74]. Dose-dependent inhibition of skin squamous cell carcinoma (SSCC) growth (*p* = 0.0012) in mice pretreated with 5 or 15 mg of C3 Complex^®^, three days prior to SSCC cells injection in each flank, and gavaged daily for 24 days. Inhibition of S6 phosphorylation, suggesting inhibition of the mTOR pathway [87]. Human gastric (MKN-45) cancer xenograft model and a mouse colon cancer (CT26) allograft model: treatment with C3 Complex^®^ in the diet (2% C3 Complex^®^ diet for 4 weeks) or by i.p. injection (50 mg/kg for 3 weeks). Significant inhibition of tumor growth and decreased percentage of MDSCs in the spleen, blood and tumor tissues, associated with reduced interleukin 6 (Il-6) level in both serum and tumor tissues [76]. Lung adenocarcinoma in mice (50mg/2.5ml/kg daily 3 or 9 days i.p.): reduction of pStat3 and the proliferative markers CycD1 and Minichromosome Maintenance Complex Component 2 (Mcm2) in murine lung tissues [77]. Murine squamous cell skin carcinoma model: topical formulation (15 mg/100 μL cream) was as effective as oral C3 Complex^®^ (15 mg daily) in suppressing tumor growth when applied for 3 days prior to xenograft injection and continuing for another 29 days [78]. Inhibition of UV-B radiation-induced skin cancer in mice (*p* =0.01) receiving identical formulations and dosages as described [78]. No significant difference in average number of tumors per mouse whether receiving C3 Complex^®^ orally or topically [88]. Murine amelanotic melanoma (B78H1) cells injected in the flank of C57BL/6 mice (4% C3 Complex^®^ diet two weeks prior to injection of tumor cells until termination of the experiment): a markedly decreased tumor volume in curcumin-treated animals at day 28. The miRNA expression signature in tumors was substantially altered, with mmu-miR-205-5p being over 100 times increasingly expressed in treated vs. control tumors [51]. Photopreventive effect with respect to UVB-induced epidermal hyperplasia in mice (C3 Complex^®^: 15 mg/kg for 5 days a week for 2 weeks) [79]. Inhibition of proliferation of tongue squamous cell carcinoma OSC19 exposed to Fibroblast Growth Factor-2 (FGF2) [82]. Reduced number of oral lesions in mice exposed to 4NQO when applied locally and systemically (C3 Complex^®^: 15 mg daily for 4 weeks, both treatments) [82]. Azoxymethane-dextran sulfate sodium (AOM-DSS) induced colitis-associated colon cancer in mice fed a C3 Complex^®^ diet 2% (*wt*/*wt*) from 5 weeks of age until the end of the 12th week. The decrease in tumor incidence and tumor multiplicity by C3 Complex^®^ was statistically significant. C3 Complex^®^ administration reversed the gene expression patterns modified by AOM + DSS, especially for genes in anti-inflammatory and anti-oxidative pathways clusters through changes of DNA methylation [89]. Mesothelioma xenograft tumor model in mice (40 mg/kg of C3 Complex^®^ i.p. daily for 4 weeks): delayed tumor growth by reducing angiogenesis and increasing apoptosis in combination with Bioperine [83].	A phase I clinical trial (*n* = 15) involving patients with advanced colorectal cancer for which no additional conventional therapies were available: curcumin and its metabolites were detected in plasma and urine in patients taking 3.6 g of C3 Complex^®^ daily for 4 months. These patients had a decrease of an inducible prostaglandin E2 (PGE2) in blood samples (indicative of biological activity and systemic pharmacological properties), irrespective of curcumin’s measurable presence/absence in plasma [90]. Pilot trial (*n* = 12; patients with advanced colorectal cancer presented with hepatic metastases; 0.45–3.6 g of C3 Complex^®^ daily, 1 week prior to surgery): low nanomolar levels of curcumin and its conjugates were found in the peripheral or portal circulation. Only the trace levels of metabolic reduction products were detected in normal liver tissue from one patient receiving 3.6 g of C3 Complex^®^ daily. Levels of malondialdehyde (MDA)-DNA adduct, which reflect oxidative DNA changes, were not decreased in post-treated normal and malignant liver tissue when compared to pretreatment samples [50]. Twelve patients with confirmed colorectal carcinoma. Giving C3 Complex^®^ capsules of 3.6, 1.8, or 0.45 g daily for 7 days pre-surgery: measurable amount of curcumin conjugates in colon tissue (both normal and cancer) after oral intake of a daily dose of 3.6 g C3 Complex^®^, but lack of quantifiable curcumin in plasma [91]. Nonrandomized, open-label, phase II trial (*n* = 25; patients with advanced pancreatic cancer; 8 g C3 Complex^®^ orally/daily until disease progression, with restaging every 2 months): high tolerability to daily intake of 8 g; despite limited absorption, stable level of conjugated curcumin in plasma was achieved on the day 3. Some improvements were described in four study participants. [92]. The single-blind, cross-over pilot study: Patients suffering from monoclonal gammopathy of undefined significance (MGUS, *n* = 26; oral intake, 4 g daily for 6 months): decrease of excessive paraprotein load (≥20 g/L) in 5/10 patients observed. In addition, 27% of patients on C3 Complex^®^ had a > 25% decrease in urinary *N*-telopeptide of type I collagen [93]. A clinical study (*n* = 100 participants; 25 control subjects and 75 patients with several diagnoses related to oral cavity (leukoplakia, submucous fibrosis, oral lichen planus); daily intake 1 g for maximally 218 days): increased level of vitamins C and E in saliva, decreased level of markers of oxidative stress malondialdehyde and 8-Hydroxy-2′-Deoxyguanosine (8-OHdG). The size of lesions significantly decreased [94]. Phase IIa clinical trial including forty-one patients (smokers) who completed the trial which explored potential influence of curcumin on ACF (daily doses: 2 and 4 g for 22 and 19 patients respectively, for 30 days). There was a significant decrease of rectal ACFs (17.8 ± 2.0, baseline, vs. 11.1 ± 2.8, postintervention; *p* < 0.005) in patients who were taking a higher daily dose (4 g). The decrease of PGE2 and 5-hydroxyeicosatetraenoic acid (5-HETE) was not recorded [38]. Randomized, double-blind placebo-controlled cross-over study including 36 patients suffering from MGUS or smoldering multiple myeloma (SMM): one group received 4 g C3 Complex^®^ and the other 4 g placebo, crossing over at 3 months. At completion of the 4 g arm (completed by 28 patients), patients entered an open-label, 8 g dose extension study for 3 months (completed by 18 patients). Based on numerous parameters measured, C3 Complex^®^ was shown to have a potential to benefit some but not all patients with MGUS or SMM [95]. A clinical pilot study (*n* =28; colorectal cancer patients undergoing colorectal endoscopy or surgical resection; daily intake of 5 × 470 mg orally for 2 weeks): curcuminoides and their metabolites were detectable in plasma of only 4 patients and urine samples of all patients, as well as in the colon mucosa of few (not all) patients who underwent colorectal endoscopy or surgical resection [24]. Clinical study (8 healthy volunteers and 15 head and neck squamous cell carcinoma (HNSCC) patients with newly diagnosed malignant tumors of the oral cavity, oropharynx, hypopharynx or larynx): self-administration of mouth dissolving microgranular formulation of C3 Complex^®^ for 10 min; 3–4 weeks regimen of 2 × 4 g daily. Only FGF-2 was significantly decreased in post-treatment tumor samples in 7 out of 11 patients when compared to evaluable matched pre-treatment tumor samples (*p* =0.0261). The decrease of FGF-2, Granulocyte-Macrophage Colony-Stimulating Factor (GM-CSF) and IL-17 in serum was significant [39]. **Adverse Events:** Two types of gastrointestinal AEs were reported by patients, which were probably related to curcumin consumption. Diarrhea occurred in two patients receiving 0.45 g and 3.6 g of C3 Complex^®^ daily. One patient consuming 0.45 g C3 Complex^®^ daily and one patient consuming 3.6 g C3 Complex^®^ daily developed diarrhea (grades 1 and 2, respectively). Nausea occurred in one patient consuming 0.9 g C3 Complex^®^ daily (toxicity grade 2). There was a rise in serum alkaline phosphatase in 4 patients (grade 1 and 2 toxicity) and increase of serum lactate dehydrogenase to >150% of pretreatment values in three patients [90]. Only one participant reported abdominal pain, bloating, nausea and diarrhea [24]. Gastrointestinal disturbances (diarrhea and distension, gastroesophageal reflux) were present in 25 participants (61%). The grade of toxicity was 1 and 2 [38].
**COMBINED**		
**Cell Culture Models**	**Animal Models**	**Clinical Studies**
Enhancement of the effect of docetaxel in HeyA8 andSKOV3ip1 ovarian carcinoma cell lines and inhibition of Tumor Necrosis Factor-Alpha (TNF-α)-mediated NF-κB activation [84]. Inhibits proliferation and potentiates the apoptotic effects of gemcitabine. Strong inhibition of constitutive NF-κB activation in four pancreatic cancer-derived cell lines (BxPC-3, MIA PaCa-2, Panc-1, Mpanc-96) [96]. C3 Complex^®^ potentiated thalidomide- and bortezomib-induced apoptosis (25% to 85% and 10% to 75%, respectively) of multiple myeloma cells and amplified inhibitory effect of bortezomib and thalidomide on NF-κB activation [72].	Several ovarian carcinoma cell lines xenografts in nude mice. C3 Complex^®^: oral gavage of 500 mg/kg for maximum 6 days, starting one week after inoculation. The combination of C3 Complex^®^ and docetaxel (35 μg) had the greatest efficacy in reduction of tumor burden in SKOV3ip1 and HeyA8 models (96%, *p* < 0.001 and 77%, *p* = 0.002, respectively). The same combination reduced the tumor mass by 66% beyond docetaxel monotherapy (*p* = 0.01) [84]. Orthotopic model of pancreatic cancer (athymic nu/nu mice injected with MiaPaca) combination of C3 Complex^®^: 1 g/kg, once daily p.o., gemcitabine: 25 mg/kg, twice weekly by i.p. injection and gemcitabine + C3 Complex^®^ for 4 weeks) potentiates antitumor activity of gemcitabine through suppression of proliferation, angiogenesis and inhibition of NFκB-regulated genes [96]. Human multiple myeloma U266 model xenograft in mice: combination of C3 Complex^®^ (1 g/kg, orally, daily) and bortezomib (0.25 mg/kg, 100 μL, weekly) for up to 20 days: potentiates the effect of bortezomib recorded as decreased tumor volume (control vs. curcumin + bortezomib *p* < 0.001; bortezomib vs. curcumin + bortezomib *p* < 0.001) [72]. Spheroids obtained from human colorectal cancer samples were exposed to 5 µM curcumin, 2 µM oxaliplatin + 5 µM 5-FU and 5 µM C3 Complex^®^ + 2 µM oxaliplatin + 5 µM 5-FU for 2 weeks. The triple combination significantly downregulated expression of pluripotent stem cell markers OCT3-4, alpha-fetoprotein (AFP) and Forkhead Box Protein A2 (FOXA2) at 24 h, and Nanog, Orthodenticle Homeobox 2 (OTX2) and Vascular Endothelial Growth Factor Receptor 2 (VEGFR2) at 72 h. Enhancement of anti-proliferative and pro-apoptotic effects in a portion of patient-derived explants was observed, associated with reduced expression of stem cell-associated markers by the addition of curcumin to oxaliplatin/5-FU [41].	An open-labeled Phase II trial for 17 patients with previously untreated locally advanced or metastatic adenocarcinoma of the pancreas. Combined treatment, gemcitabine (1000 mg/m^2^ intravenously weekly) with C3 Complex^®^: (2 × 4 g daily), for 1 week to 12 months (median 2.5 months). The effect was reported for 11 patients: partial response in one patient during 7 months, stable disease from 2–12 months in four patients, tumor progression in six patients [97]. A phase I/II study of gemcitabine-based chemotherapy plus C3 Complex^®^ for patients with gemcitabine-resistant pancreatic cancer (*n* = 21; 8 g oral C3 Complex^®^: daily in combination with gemcitabine-based chemotherapy): combination therapy with gemcitabine-based chemotherapy is safe and feasible [98]. The phase I dose escalation study (*n* = 12) of patients with colorectal liver metastases: 500 mg (1 capsule) of oral curcumin C3 Complex^®^: daily, 7 days prior to the scheduled chemotherapy (up to 2 g daily): safe and tolerable adjunct to folinic acid/5-fluorouracil/oxaliplatin chemotherapy (FOLFOX); by treatment end, 81.8% patients had no concerns regarding side-effects from C3 Complex^®^ [41]. Randomized phase IIa trial to assess safety, efficacy, quality of life, neurotoxicity, curcuminoids and C-X-C motif chemokine ligand 1 (CXCL1) in patients with metastatic colorectal cancer receiving FOLFOX compared with FOLFOX + 2 g oral C3 Complex^®^/d (CUFOX): combination of C3 Complex^®^: and FOLFOX chemotherapy is safe and tolerable. There was no significant difference between arms for quality of life or neurotoxicity. Curcumin glucuronide was detectable at concentrations >1.00 pmol/mL in 15 of 18 patients receiving CUFOX. Curcumin did not significantly alter CXCL1 over time [40]. **Adverse Events:** Kanai et al. observed neutropenia (38%; grades 3–4) and fatigue (10%; grades 3–4), which were not attributed to C3 Complex^®^, but to the gemcitabine-based chemotherapy or disease progression [98]. There is a possibility that some side effects developed in patients receiving C3 Complex^®^ and FOLFOX were attributable to curcumin: diarrhea was the most frequently reported event (in 66.7% of patients) [41]. The most common AE reported as possibly or probably related to C3 Complex^®^ was diarrhea, nausea, oral mucositis and constipation [40]. Gastrointestinal toxicity manifested as abdominal fullness and pain in 7 patients. In 5/7 patients the adverse event was intractable (Grade 3) and resulted in cessation of C3 Complex^®^ administration. In one of these patients, the duodenal peptic ulcer exacerbated. Two patients experienced Grade 2 abdominal pain. In these patients, the daily dose was reduced and continued on a reduced dose of 4 g/day of curcumin, after 2 and 6 weeks of full treatment, respectively. Mild hematological toxicity was observed in four patients (neutropenia grade 2 in two patients; 2 thrombocytopenia of grade 1 and 2). It was concluded that a daily dose of 8 g of C3 Complex^®^ may be above the maximum tolerated dose when combined with gemcitabine [97].

* The possibility that Bioperine was applied in more than one study [83] cannot be excluded.

**Table 3:** Effects of Meriva^®^ obtained in vitro, in vivo and in clinical trials.

Meriva^®^		
**MONO**		
**Animal Models**		**Clinical Studies**
Xenograft study of a mammary gland tumor cell line ENU1564 in athymic nude mice (*n* = 18): Meriva® group was fed daily with Tekled 2019 containing 6% Meriva®. Decreased number of metastatic foci in the lung (*p* < 0.041) in comparison to the control and curcumin groups. Reduced expression of matrix proteinase 9 (MMP-9), but not Vascular Endothelial Growth Factor (VEGF), was shown in Meriva® fed animals’ tumors [99]. BALB/c mice injected with triple-negative breast cancer cell line 4T1 (only 3 animals per group): Cryoablation plus Meriva® (but not Meriva® alone) strongly reduced tumor growth and the number of lung metastases. Overall survival was improved in animals treated with combination of Meriva® and cryoablation. When 10 mg of Meriva® (every 3 days for 14 days starting when tumors are 1–1.5 cm in diameter) was given, there was a decrease in Il-6 production and number of metastatic foci [100].		A controlled study that included 160 cancer patients (terminal patients excluded) with various solid tumors: one tablet containing 0.5 g Meriva® was given three times daily between the 4th and 16th weeks from surgery, for at least 60 consecutive days. The observational framework: 4 months, starting from the day after their first cycle of chemotherapy or radiotherapy. Semi-quantitative assessment of side effects: statistically significantly improved (*p* < 0.05) plasma oxidative status between Meriva® group and control group [101].
**COMBINED**		
**Cell Culture Models**	**Animal Models**	**Clinical Studies**
In combination with oxaliplatin decreases proliferative capacity of oxaliplatin-resistantHCT116 colorectal cell line, irrespective of their Tumor Protein P53 (TP53) status [102].	Xenograft colorectal cancer (HCT116) in nude mice: 1.13% Meriva® mixed with a standard diet for 21 days. The decrease of tumor volume was 53%, 35% and 16% (Meriva® + oxaliplatin vs. Meriva® vs. oxaliplatin). No significant difference in DNA platinating ability but significantly increased cleaved-caspase-3-positive cells by 4.4-fold (*p* <0.05) when Meriva® + oxaliplatin were applied [102].	A pilot, product evaluation registry study (*n* = 33; patients with benign prostate hyperplasia; 2 × 0.5 g of Meriva^®^/day for at least 24 weeks + “BSM” (Best Standard Management) vs. the BSM group (*n* = 28)): the addition to the standard treatment contributed to the reduction of signs and symptoms of the disease without causing any significant additional side effect. Statistically significant decrease of PSA, *p* < 0.025 [103]. A randomized, double-blind, placebo-controlled trial (*n* = 80; patients with solid tumors receiving a conventional chemotherapy; Meriva®: 180 mg/day for 8 weeks). Suppression of some (TNF-α, calcitonin gene-related peptide (CGRP), monocyte chemoattractant protein 1 (MCP-1): *p* < 0.001), but not all (IL-8) measured systemic inflammation markers. An improvement of quality of life was reported [104]. Single-center, single-arm prospective phase II trial (*n* = 52; advanced pancreatic cancer; gemcitabine 10 mg/m^2^/min infused over 100 min on days 1, 8, 15 and Meriva^®^ 2 g/day continuously, each cycle repeated every 28 days). There is no strong evidence on improved safety and efficacy of gemcitabine when combined with Meriva® in patients suffering from advanced pancreatic cancer [105]. **Adverse Events:** Mild gastrointestinal side effects (not specified) occurred in 8 patients [104].

**Table 4:** Effects of Lipocurc™ obtained in vitro, in vivo and in clinical trials.

Lipocurc™		
**Cell Culture Models**	**Animal Models**	**Clinical Studies**
Significantly higher activity when compared to curcumin: reduction in the IC_50_ in A549 lung cancer cells [106]. Cell-type-specific dose-dependent response to Lipocurc™ and curcumin was recorded as significantly higher in human osteosarcoma cell lines than in canine cell lines originating from various cancers. Parameters explored: cellular viability, migration and formation of tubes [107].	Xenograft tumor growth of A549 non-small cell lung cancer cells in athymic nude mice (20 mg/kg s.c. twice a week for seven weeks). Regression of tumor volume was significant and was associated with a strong decrease of NFκB-p65, Ki-67 and Annexin A2, as recorded by immunohistochemistry [106]. Naturally occurring various canine lung cancers (initially included 11 dogs; Lipocurc™ infusion administered four times per week, 10 mg/kg): the study reports mixed results. Four out of six dogs experienced stable disease, and no radiographic responses were detected [107].	Phase I, single-center, open-label study in patients with advanced metastatic tumors (*n* = 32): maximum tolerated dose in patients with metastatic tumors was determined (300 mg/m^2^ over 6 h) and recommended as starting dose for testing Lipocurc™ in clinical cancer trials. The study is not conclusive with respect to the primary disease [33]. Cancer patients: Analysis of the impact of co-medication on infusion rate normalized plasma levels and comparison of the plasma levels between cancer patients and healthy individuals: either co-medications or health status, or both, can impact the pharmacokinetics of Lipocurc™ infusion in cancer patients [108]. **Adverse Events:** Among143 AEs recorded in 30 patients (93.8%), only 11 AEs (in 9 patients) were considered definitely or probably related to the treatment itself. These included facial edema, anemia, hemolysis, mild gastrointestinal symptoms and a few others. It was difficult to make a clear distinction between symptoms and complications associated with the advanced primary disease and the treatment itself [33].

**Table 5:** Effects of LongVida^®^ obtained in vitro, in vivo and in clinical trials.

LongVida^®^	
**Cell Culture Models**	**Clinical Studies**
Effective in human and mice glioblastoma cell lines, where it was shown to have significantly stronger response than with natural curcumin. Parameters tested: cellular viability, DNA fragmentation and apoptosis.A statistically significant increase of P53 and decrease of c-Myc [109].	Safety and pharmacokinetics study (*n* = 11; patients with osteosarcoma; doses of 2, 3 and 4 g): in both healthy individuals and osteosarcoma patients, high interindividual variability in pharmacokinetics and nonlinear dose dependency was observed; overall, good tolerability was noted in both groups [53]. A randomized clinical trial (*n* = 30; oral submucous fibrosis patients): the total daily dose of 2 g was given for 3 months duration and follow-up was done for 6 months: promising in the treatment of oral submucous fibrosis with respect to tested parameters [110]. **Adverse Events:** Pigmentation of the tongue and teeth [110].

**Table 6:** Effects of Theracurmin™ obtained in vitro, in vivo and in clinical trials.

Theracurmin™		
**MONO**		
**Cell Culture Models**	**Animal Models**	**Clinical Studies**
Inhibition of proliferation and increased apoptosis of three different esophageal cancer cell lines (50 µM for 48 h). Modulation of the cytokine profile of activated T cells toward a profile that negatively affects tumor cell growth and migration (reduced secretion of TNF-α, and four interleukins: IL-8, IL-6, IL-10 and IL-1β) [111]. Efficiently reduces viability and inhibits the growth of human prostate and bladder cancer cell lines via induction of apoptotic cell death and cell cycle arrest [112]. Inhibition of cell proliferation and induction of apoptosis of human prostate cancer cell lines LNCaP and 22Rv1 in a dose-dependent manner. Strong influence on the proteins involved in steroidogenesis (decrease of Cytochrome P450 Family 11 Subfamily A Member 1 (CYP11A1) and Hydroxy-Delta-5-Steroid Dehydrogenase, 3 Beta- and Steroid Delta-Isomerase 2 (HSD3B2), increase of Cytochrome P450 Family 17 Subfamily A Member 1 (CYP17A1)), supporting the decrease of testosterone production [113]. Induction of cytotoxic and antiproliferative effects in several cell lines originating from esophageal squamous cell carcinoma (ESCC). Theracurmin™associated with increase of ROS and activation of the NRF2–NMRAL2P–NQO1 (Nuclear Factor (Erythroid-Derived 2)-Like 2-NMRA Like Redox Sensor 2-NAD(P)H Quinone Dehydrogenase 1) axis [55]. Decreased activity of NF-κB and its targets in two colon cancer cell lines (HCT116 and DLD1) [114]. Reduced viability of castration-naïve and castration-resistant prostate cancer-derived cell lines with conditional Pten (Phosphatase and Tensin Homologue) knock-out in a time- and dose-dependent fashion [115].	Prostate tissues from the transgenic adenocarcinoma of the mouse prostate (TRAMP) model were obtained after 1-month oral administration of 200 mg/kg/day Theracurmin™. Decreased testosterone levels in the prostate tissues were observed (*p* < 0.01), but there was no influence on the serum testosterone level [113]. Hairless SCID male mice subcutaneously injected with 1.5 × 106 TE-11R cells, randomly assigned to three groups and fed with control diet, curcumin or Theracurmin™ supplemented diet from day 0 to day 70. Theracurmin™ group had greater reduction of tumor mass (43%) compared to curcumin group (11%) on day 70. Patient-derived ESCC xenografted tumors in C57BL/6 male mice who were drinking Theracurmin™-containing water from day 21 to day 70; 5000 ppm. A strong suppression of tumor growth, especially when combined with the NQO1 inhibitor [55]. Treatment with 500 ppm Theracurmin™ for 8 weeks inhibits development of polyps which were <0.5 mm or 0.5 < 0.9 mm in diameter (*p* < 0.05 vs. control group), in Apc mutant mice. However, there was no significant decrease of the total number of polyps. Inhibition of proliferation (proliferating cell nuclear antigen; PCNA) and inflammation-related (Mcp-1 and Il-6 mRNA) factors [114]. Mouse model of Pten-deficient prostate cancer: sixteen weeks of dietary supplementation of 76 mg/kg/day or 380 mg/kg/day nanoparticle Theracurmin™ did not influence tumor burden, but mice fed with high-dose Theracurmin™ had lower cancer cell proliferation rates at 12- and 16-, but not at the 20-week time [115].
**COMBINED**		
**Cell Culture Models**	**Animal Models**	**Clinical Studies**
Combination with Erlotinib augmentatively reduces cell viability in four human lung cancer cell lines (*p* < 0.001; H1299, H460, A549 and PC9), partly through IkappaB expression [116]. Knockdown of NQO1 in TE-11R esophageal carcinoma cell line increased susceptibility to Theracurmin™. Conversely, overexpression of NQO1 in TE-11R cells resulted in a decrease of 8-OHdG-indicated oxidative damage after THC treatment and was associated with resistance to THC treatment [55].	Human lung adenocarcinoma (PC9) tumors in mice: animals were receivingTheracurmin™(100 mg/kg by oral gavage, every 2 days for 2 weeks), administratedwith or without Erlotinib.The combined treatment significantly attenuated the tumor weight (*p* < 0.01) [116]. Patient-derived xenograft (PDX) esophageal squamous cell carcinomas in hairless SCID male mice. Animals were given either pure water or Theracurmin™-containing water (5000 ppm) from day 21 to day 70. The combination of THC and NQO1 inhibitor resulted in a significant inhibition of PDX tumor growth compared with that after control or monotherapy (inhibition at day 70: NQO1 inhibitor 1.9%, THC 40.7%, THC plus NQO1 inhibitor 72.6%) [55].	Phase I study (*n* = 16; pancreatic or biliary tract cancer patients who failed standard chemotherapy): water solution of Theracurmin™ containing 200 mg of THC as a starting dose which swas safely escalated to 400 mg, orally administered every day with standard gemcitabine-based chemotherapy. A repetitive, systemic exposure to high concentrations of curcumin achieved by Theracurmin™ did not increase the incidence of adverse events in cancer patients receiving gemcitabine-based chemotherapy. The changes of NF-κB activity (in PBMCs), IL-6 and TNF-α (in plasma) were not demonstrated [117].
